# An observed, prospective field study to evaluate the performance and acceptance of a blood-based HIV self-test in Canada

**DOI:** 10.1186/s12889-021-11418-z

**Published:** 2021-07-18

**Authors:** Richard A. Galli, Jason M. Lo Hog Tian, Michelle Sumner-Williams, Kristin McBain, Emal Stanizai, Wangari Tharao, Muna Aden, Heather Jamieson, Mark Da Silva, Anne-Fanny Vassal, Lorie Guilbault, Laurie Ireland, Kim Witges, Alexandra King, Kehinde Ametepee, Nathan J. Lachowsky, Nitika Pant Pai, Tony Mazzulli, Sean B. Rourke

**Affiliations:** 1 MAP Centre for Urban Health Solutions, Unity Health Toronto, Toronto, Ontario Canada; 2 Institute of Medical Science, University of Toronto, Toronto, Ontario Canada; 3 Women’s Health in Women’s Hands, Toronto, Ontario Canada; 4 Hassle Free Clinic, Toronto, Ontario Canada; 5 Clinique Medicale L’Actuel, Montreal, Quebec, Canada; 6 Nine Circles Community Health Centre, Winnipeg, Manitoba Canada; 7 University of Saskatchewan, Saskatoon, Saskatchewan, Canada; 8 Community Based Research Centre, Vancouver, British Columbia Canada; 9 University of Victoria, Victoria, British Columbia Canada; 10 McGill University, Montreal, Quebec, Canada; 11 Public Health Ontario Laboratory, Toronto, Ontario Canada; 12 Department of Psychiatry, University of Toronto, Toronto, Ontario Canada

**Keywords:** HIV, Blood-based self-test, Accuracy, Usability, Acceptance

## Abstract

**Background:**

Self testing for HIV is a targeted intervention with the potential to increase the access, uptake and frequency of HIV testing and more effectively reach the undiagnosed, especially in priority populations. The objectives of this study were to (1) evaluate the INSTI HIV self-test performance compared with laboratory reference testing, (2) document if intended users can perform the steps to use the HIV self-test device, and (3) document if intended users can successfully interpret contrived positive, negative, and invalid results. Study was intended to be submitted to Health Canada for review for regulatory approval purposes.

**Methods:**

The study used a cross-sectional design and recruited consenting adults who were representative of intended users of HIV self-testing from four community sites across Ontario, Québec, and Manitoba between August 2019 and March 2020. The results of the observed HIV self-test were compared with results of the Abbott Architect HIV Ag/Ab Combo test. Usability outcomes for critical (e.g., lancing finger, blood droplet into bottle, shaking bottle four times) and noncritical self-test procedure steps were also determined.

**Results:**

Overall, 77% (*n* = 522) of participants were between 18 and 45 years of age, 61% (*n* = 410) were male, 71% (*n* = 480) had some college or more education, and 45% (*n* = 307) were employed; identity for race and ethnicity: Caucasian (44%; *n* = 296), African, Caribbean or Black (17%; *n* = 113), Indigenous [First Nations, Métis or Inuit] (14%; *n* = 95), Asian (16%; *n* = 106), Latin American (7%; *n* = 46). Primary performance analysis on 678 completed HIV self-tests revealed a positive percent agreement of 100% (5/5, 95% CI: 43.6–97.0%) and a negative percent agreement of 99.5% (614/617, 95% CI: 98.6–99.8%) with the comparator method. The overall percent agreement of results interpretation between participant and observer was 93.5% (*n* = 633). For the 708 participants who took part in the usability study, the average success rate for steps determined to be “critical” for successful completion of the test was 92.4%. 97% (*n* = 670) of participants found the instructions easy to follow, and 95% (*n* = 655) of participants indicated that they would use the test again. Of the 404 participants who interpreted the strong positive, weak positive, negative, and invalid contrived results, successful interpretation ranged from 90.6% (for weak positive, *n* = 366) to 99.3% (for negative, *n* = 401).

**Conclusions:**

The addition of a regulatory-approved self-test into the Canadian HIV testing landscape could significantly increase HIV testing rates. Having a blood-based HIV self-test approved in Canada can offer an accurate, acceptable, and simple alternative to facility-based HIV testing, particularly when impacted by Coronavirus pandemic restrictions.

**Supplementary Information:**

The online version contains supplementary material available at 10.1186/s12889-021-11418-z.

## Background

Unlike other G7 countries, Canada is not seeing a reduction in the overall number of new people being diagnosed with HIV. Recent data from the Public Health Agency of Canada (PHAC) indicate that an estimated 2242 new HIV infections occurred in Canada in 2018 compared to 2165 in 2016, which is an increase of 3.6%. Approximately 13% of people living with HIV in Canada are still not diagnosed, which represents over 8000 individuals living with HIV who are unaware of their status [1]. Although Canada had endorsed the initial UNAIDS 90–90-90 target (90% diagnosed, 90% of those on treatment and in care, and 90% of those achieving viral suppression) [2], only 87% of the estimated 62,050 people living with HIV are diagnosed (1st 90 target) – indicating these 8000+ individuals who have undiagnosed HIV infection across the country may not be adequately served by or connected to our health care system. For those people diagnosed with HIV, 85% were on antiretroviral treatment (2nd 90 target), and of those, 94% had suppressed viral load (3rd 90 target) by the end of 2018 [3]. While we have reached one of three key UNAIDS targets, there are nearly 20,000 people who have not yet been diagnosed or who are not on antiretroviral therapy in Canada, which represents nearly a third of the total people living with HIV in Canada. Targeted interventions for testing, such as using HIV self-testing to reach the undiagnosed particularly amongst key and hard to reach populations, and supporting more people to manage and adhere to treatment, and achieve viral suppression are needed to achieve Canada’s original UNAIDS commitment to all three of the 90–90-90 targets and now move successfully towards ending its HIV epidemic in the next 5 years. Indeed, diagnosing HIV is critically important for reaching targets aimed at controlling the HIV epidemic [4].

In 2016, the WHO recommended HIV self-testing as an alternative to conventional facility-based testing [5]. By 2018, 59 countries had developed national policies on HIV self testing, however Canada had not finalized a national policy [6]. In many countries, self-testing for HIV is an established targeted intervention with the potential to increase the access, uptake and frequency of HIV testing, and could potentially become a high impact, low cost, and empowering alternative for those who may not otherwise test, notably for populations at high risk for HIV infection [7]. In a review on modern diagnostic technologies for HIV, the authors conclude that these novel technologies, including blood-based HIV self tests, show promise as they are associated with ease of use, high diagnostic accuracy, rapid detection, and the ability to detect HIV-specific markers [8]. There is a growing body of supporting evidence showing the acceptance and usability of HIV self-testing in various global key populations and groups [7, 9, 10], however similar study data for blood-based HIV self testing within the Canadian population had not existed prior to this study.

Three earlier studies were conducted in Montreal, Quebec with oral fluid HIV self tests in key populations and low risk populations. In these studies, HIV self-testing using oral fluid was demonstrated to be an acceptable and feasible testing solution, with data on technology innovations that can help increase testing and expedite linkages to care [11, 12]. A survey of Canadian stakeholders demonstrated that HIV self testing is desired by Canadians yet, operational models and linkage data need to be thought through [13]. Despite these favourable outcomes supporting the use of HIV self-testing, the first blood-based HIV self test has only just been approved by Health Canada on November 3, 2020 with implementation and scale-up critical as next steps.

To assess if favourable outcomes similar to those of the oral fluid self test studies conducted earlier can be achieved with a blood-based self test, this study aims to provide independent data on the INSTI HIV Self-Test (BioLytical Laboratories, Richmond, BC, Canada) performance, acceptance, and usability in the hands of intended users in Canada. The study objectives were to (1) evaluate the device performance, i.e., sensitivity, specificity, and percent agreement compared with laboratory reference testing, (2) document if intended users (non-professional and inexperienced in HIV self-testing) can successfully perform the steps to use the HIV self-test device without product demonstration, and (3) document if intended users can successfully interpret contrived strong positive, weak positive, negative, and invalid results.

## Methods

### Study design

The study used a cross-sectional design and recruited consenting adults from four community-based sexual health and medical clinics or health centres across Ontario, Québec, and Manitoba between August 2019 and March 2020. Due to the emergence of the COVID-19 infection in populations throughout Canada and related restrictions concerning clinical practice, study sites were forced to discontinue study enrolment by March 18, 2020.

### Inclusion criteria

The study was open to the English and French-speaking general public over the age of 18 who met the inclusion criteria (i.e., consented to have venous blood drawn and undergo self-testing by an investigational device) and did not meet any of the exclusion criteria (see Appendix A for full list of inclusion and exclusion criteria). No participant was excluded based on race, gender, ethnicity or sexual orientation. Participants were compensated with $50 at their initial study site enrollment and an additional $25 when they returned for their laboratory testing results.

### Study population and sample size

Self-reported HIV risk activity for the year prior to the study enrolment was collected for each participant. Recruitment occurred during routine clinic visits through use of brochures, waiting room posters, and word of mouth. The original sample size for the study was intended to be 900 individuals with unknown HIV status including a minimum of 400 “at-risk” of HIV infection, which is the number required for HIV self-test prospective studies in the published Health Canada guidelines (Health Canada, 2017).

### Study protocol

The results of the INSTI HIV Self-Test performed and interpreted by intended users were compared with results of licensed laboratory-based Comparator Methods (CM). The CM used for all sites was the HIV-1/HIV-2 combination antigen/antibody test (Abbott Architect HIV Ag/Ab Combo test, Abbott Laboratories, Abbott Park, IL) which is licensed by Health Canada and is in routine use at the central public health laboratories of each of the participating provinces. The Geenius™ HIV 1/2 Confirmatory Assay (Bio-Rad Laboratories, Marnes-la-Coquette, France) was used for positive confirmation where necessary.

After written informed consent was obtained, one venous blood sample was collected from each participant for testing at a central laboratory by the CM. Each participant then started the self-test – they self-collected a fingerstick blood specimen and performed the INSTI HIV Self-Test, including result interpretation, according to only the manufacturer’s instructions for use. Each self-test performance was directly observed by a trained healthcare professional (Observer). The Observer did not tutor or interact with the participant conducting the INSTI HIV Self-Test but noted errors and other observations about the participant’s test performance. There were 24 items developed, similar to those used in a usability assessment of multiple HIV self-test devices in South Africa [14] to capture usability information including instructions for use, pre-ST preparation, procedure for self-collecting blood sample, self-test procedure, and follow-up procedures. Half of the items (12 in total) were considered “critical” with respect to correct usability. The Observer also interpreted the HIV self-test result immediately after the participant and recorded the result separately. Lastly, a sub-set of participants who volunteered were provided the contrived “mock” result membrane cartridges individually and asked to interpret the results. All survey instruments were developed specifically for this study (see Appendix C for full survey instruments).

The provincial public health laboratories conducted CM testing on serum from venous blood samples collected from study participants and the results were provided back to the sites and subsequently to the central data coordination centre at St. Michael’s Hospital, Toronto. Each central laboratory test result was used for study device performance evaluation with the INSTI HIV Self-Test, however study participants were able to return to the clinic site at a later date to receive their laboratory testing results if desired. All clinical care decisions were based solely on the results of the standard of care HIV testing in place at each site.

### Electronic data capture

All participant data were collected by the study observers through the use of pre-loaded tablets at the study sites and stored using Qualtrics (Qualtrics, Provo, UT, USA https://www.qualtrics.com), a survey collection tool that encrypts data using Hypertext Transfer Protocol Secure (HTTPS) and enforces HTTP Strict Transport Security (HSTS). Participant data were stored on local encrypted servers in Toronto at St. Michael’s Hospital for further data analysis. Only designated research team members accessed the password-protected data from the Qualtrics servers. The survey data for each participant were coded with a corresponding unique study ID to maintain privacy and confidentiality.

### Ethics approval

This study was approved by respective Research Ethics Boards (REB) for the Ontario, Québec and Manitoba sites participating in the study: University of Toronto REB, St. Michael’s Hospital REB, Veritas IRB Québec, and University of Manitoba HREB. This study was conducted under a Health Canada approved Investigational Testing Authorization (ITA), application No. 276320, issued on December 19, 2018.

### Data analysis

All data analysis was conducted using IBM SPSS Statistics version 24. The Primary Efficacy Analysis (performance) includes calculation of positive and negative percent agreement between study participants’ self-interpreted results of the INSTI HIV Self-Test versus the 4th generation Abbott Architect test results. The proportion of study participants’ interpretation of their self-test which is confirmed by the confirmatory test algorithm, and thereby considered “true” will be confirmed following determination of study participant’s data inclusion in either the positive percent agreement (PPA) population or the negative percent agreement (NPA) population. Invalid self-test results and results indicated as “do not know” (*n* = 56) were excluded from the calculation of PPA and NPA.

The overall 95% confidence interval will be determined where: PPA = [TP / (TP + FN)] × 100, where TP (true positive) is positive self-test in agreement with positive Architect test, and FN (false negative) is negative self-test discordant with positive Architect test; and NPA = [TN / (TN + FP)] × 100, where TN (true negative) is negative self-test in agreement with negative Architect test, and FP (false positive) is positive self-test discordant with negative Architect test.

As a secondary analysis, study participants’ self-interpreted results were compared with the self-test results interpreted by the observer.

## Results

### Study participants

Due to the impact of COVID-19 on restrictions for facility visits, a total of 767 participants were recruited over the study period instead of the 900 that were targeted for enrollment. Of the 767 participants that started the process, 89 participants were excluded from the Primary Efficacy Analysis for reasons including failure to provide a venous blood sample or failure to provide a self-test result (see Appendix B for full details of excluded participants). Recruited participant numbers varied across the performance, usability and mock results interpretation arms of the study as not all recruited participants completed all elements.

Table 1 shows the demographic profile of participants included in the performance analysis. Over 75% (*n* = 522) of the sample was between the ages of 18 and 45 years of age. Over 60% (*n* = 410) identified as “male” and 37% (*n* = 247) as “female” with approximately 3% (*n* = 20) choosing “other”, including transgender, non-binary, queer, and Two Spirit gender identities. In terms of ethno-racial composition, approximately 44% (*n* = 296) identified as “White”, 17% (*n* = 113) as “Black”, 14% (*n* = 95) as “First Nations, Métis or Inuit”, 16% (*n* = 106) as “Asian”, 7% (*n* = 46) as “Latin American”, and the remaining 2% (*n* = 22) as “other”. In terms of educational achievement, approximately 27% (*n* = 184) of the sample completed High School, 23% (*n* = 156) completed College, and 48% (*n* = 322) a University degree or higher. Less than 5% (*n* = 30) indicated having any kind of reading or writing impairment. Approximately 45% (*n* = 307) were employed, 23% (*n* = 153) were unemployed, 13% (*n* = 87) were students, 2% (*n* = 16) were retired, and 17% (*n* = 113) preferred not to answer.

**Table 1:** Demographic Profile of Participants included in the Primary Efficacy Analysis (*N* = 678*)

	%
**Age Range [*n*** **= 678]**	
18–25 (*n* = 139)	20.5%
26–35 (*n* = 277)	40.9%
36–45 (n = 106)	15.6%
46–55 (*n* = 95)	14.0%
> 55 (*n* = 61)	9.0%
**Gender [*n*** **= 677]**	
Male (n = 410)	60.6%
Female (n = 247)	36.5%
Other (*n* = 20)	2.9%
**Race/Ethnicity [*n*** **= 678]**	
White (*n* = 296)	43.7%
Black (*n* = 113)	16.7%
First Nation, Metis, Inuit (*n* = 95)	14.0%
South Asian (*n* = 26)	3.8%
Southeast Asian (*n* = 45)	6.6%
Arab/West Asian (*n* = 35)	5.2%
Latin American (*n* = 46)	6.8%
Other - includes mixed ethnicity (n = 22)	3.2%
**Highest Education Level [*n*** **= 678]**	
Primary (*n* = 16)	2.4%
Secondary (High School) (*n* = 184)	27.1%
College (*n* = 156)	23.0%
University or Higher (*n* = 322)	47.5%
**Reading/Writing Impairment [*n*** **= 674]**	
Yes (n = 30)	4.5%
No (*n* = 644)	95.5%
**Dominant Hand [n = 677]**	
Right (*n* = 602)	88.9%
Left (*n* = 75)	11.1%
**Visual Status (Use of reading glasses) [n = 678]**	
Yes (*n* = 373)	55.0%
No (*n* = 305)	45.0%
**Participant is Pregnant [n = 674]**	
No (*n* = 672)	99.7%
Yes (*n* = 2)	0.3%
**Employment Status [*n*** **= 676]**	
Employed (*n* = 307)	45.4%
Unemployed (*n* = 153)	22.6%
Student (*n* = 87)	12.9%
Retired (*n* = 16)	2.4%
Prefer not to answer (*n* = 113)	16.7%
**Self-Reported Medical Conditions [*n*** **= 224]**	
Diabetes (*n* = 23)	10.3%
Hypertension (*n* = 39)	17.4%
Visual impairment (*n* = 58)	25.9%
Existing/Recent sexually transmitted diseases (*n* = 20)	8.9%
Other (*n* = 84)^a^	37.5%
**Self-Reported HIV Status [n = 677]**	
Negative status (*n* = 540)	79.8%
Unknown/Never been tested (*n* = 137)	20.2%
Positive status (*n* = 0)	0.0%
**Experience with HIV testing [n = 674]**	
Yes (*n* = 500)	74.2%
No (*n* = 174)	25.8%
**Self-Reported Risk Category [*n*** **= 611]**^**b**^	
Unprotected sex with men (*n* = 185)	30.3%
Unprotected sex with women (*n* = 108)	17.7%
Multiple sexual partners (*n* = 181)	29.6%
Injection drug user (*n* = 51)	8.3%
Born to HIV positive mother (*n* = 3)	0.5%
Sexual partner is HIV positive (*n* = 26)	4.3%
Sexual partner is a bisexual male (*n* = 46)	7.5%
Other (*n* = 11)^c^	1.8%
**Self-Reported Risk [*n*** **= 678]**^**d**^	
High risk (*n* = 600)	88.5%
Low/Unknown risk (*n* = 178)	11.5%

*Category totals below *N* = 678 reflect missing data

^a^Common “other” medical conditions include prediabetes, mental illness, Hepatitis C, asthma, hypothyroidism, and cancer

^b^Risk categories are mutually exclusive; participants who self-reported multiple risks were put into the highest risk category

^c^Common “other” risk categories include “protected sex” and “sex with partner”

^d^“High risk” contains participants who self-reported one or more risk category, “low/unknown risk” contains participants who self-reported “other” or no risks

In terms of self-reported HIV status, 20% (*n* = 137) indicated that their status was unknown or had never been tested, while 80% (*n* = 540) indicated that they were HIV-negative. When asked about HIV testing, 74% (*n* = 500) indicated having prior experience with testing. When evaluating self-reported risk category for HIV, 600 participants (89%) were considered “high risk” for HIV infection. For specific self-reported sexual behaviours in the year prior to survey, 30% (*n* = 185) identified as having condomless sex with men while 18% (*n* = 108) had condomless sex with women. Approximately 30% (*n* = 181) of participants indicated having multiple sexual partners in the past year, 26 individuals (4%) indicated that their sexual partner was living with HIV, and 51 participants (8%) indicated having ever injected drugs.

### Primary efficacy (performance) analysis

After removing the 89 subjects who met the exclusion criteria from the 767 recruited participants, the primary efficacy analysis on the 678 who completed INSTI HIV Self-Test revealed a positive percent agreement of 100% (5/5, 95% CI: 43.6–97.0%) and a negative percent agreement of 99.5% (614/617, 95% CI: 98.6–99.8%) when comparing the valid self-tester results to the Abbott Architect (see Table 2 for full comparison of participant and laboratory test results and Table 3 for PPA and NPA calculations). Of the 6 HIV previously undiagnosed participants living with HIV that were confirmed by the Abbott Architect and Geenius comparator methods, 5 were positive with the self-test and 1 was invalid due to a low volume of fingerstick blood that was used in the INSTI self-test procedure. The overall percent agreement between participant and observer for the self-test results was 93.5% (*n* = 633) (see Table 4 for full comparison of participant and observer interpretations of HIV-ST results). The overall “invalid” rate for the HIV Self-Test was 5.6% (*n* = 38).

**Table 2:** Agreement between participant interpreted HIV-ST result and Laboratory Result (*n* = 678)

	Lab Result		
Participant Interpreted Result	Negative	Positive	Total
Negative	614	0	614
Positive	3	5	8
Do not know/Not sure	18	0	18
Invalid/test did not work	37	1	38
Total	672	6	678

**Table 3:** Positive and Negative Percent Agreement with Laboratory Results (Abbott Architect) based on 678 completed HIV Self-Tests

True Positive	6
False Negative	0
True Negative	669
False Positive	3
Positive Percent Agreement^a^	5/5 = 100% (95% CI: 43.6–97.0%)
Negative Percent Agreement^b^	614/617 = 99.5% (95% CI: 98.6–99.8%)

^a^One additional HIV positive subject (ON02–015) had an invalid self-test result due to wiping the fingertip on the rim of bottle 1 then dropped the bottle and spilled most of the contents before adding the remainder to the membrane unit. Abbott Architect results were positive. This invalid was not used in the calculation

^b^Participants with invalid self-test results (*n* = 37) and who indicated “do not know” for the self-test result (n = 18) are not included in the calculation

**Table 4:** Agreement between self-tester interpreted result and observer interpreted result (_N_ = 678)

	Observer Interpreted Result				
Participant Interpreted Result	Negative	Positive	Do not know/Not sure	Invalid/Test did not work	Total
Negative	602	0	6	6	614
Positive	2	5	0	1	8
Do not know/Not sure	8	0	4	6	18
Invalid/test did not work	15	0	1	22	38
Total	627	5	11	35	**678**

### Usability assessment analysis

From the total of 767 study participants, 59 subjects withdrew after consenting, did not have a venous blood sample collected or did not complete the self-test, leaving 708 available for analysis with the usability assessment. Overall, the average for all expected outcomes was 91.8% and for the 12 critical items, the average was 92.4% (see Table 5 for full breakdown of usability questions). Despite as instructed by the kit instructions for use, only 47.2% (*n* = 334) of participants were observed to have washed and dried their hands. Just over 92% (*n* = 650) of participants were able to lance their finger correctly, and 88.7% (*n* = 627) were able to form a blood droplet, but there was a considerably lower number of participants (81.2%; *n* = 575) who were able to get the blood droplet to fall directly into Bottle 1.

**Table 5:** Usability Assessment (*n* = 708)

Question	Yes		No	
	%	n	%	n
**Instructions for Use (IFU)**				
Did the study participant read/use the Instructions for Use (IFU)?	99.4%	700	0.6%	4
If yes, was the Instructions for Use (IFU) read before the test?	94.6%	668	5.4%	38
Was it referred to during the test process?	99.4%	695	0.6%	4
**Pre-Test**				
Did the study participant wash and dry their hands as instructed in the Instructions for Use (IFU)?	47.2%	334	52.8%	374
Was it difficult for the study participant to remove the test device from the pouch?	12.5%	88	87.5%	616
Was the study participant able to remove the cap of Bottle 1?	99.0%	696	1.0%	7
Did the study participant twist the tip of the lancet off?	99.7%	705	0.3%	2
Did the study participant rub his/her finger correctly (up and down/vertical motion)?^a^	88.8%	626	11.2%	79
**Blood Draw**				
Was the study participant able to lance his/her finger correctly?^a^	92.1%	650	7.9%	56
Was the study participant able to form a blood droplet?	88.7%	627	11.3%	80
Was the study participant able to get the blood droplet to fall into Bottle 1?^a^	81.2%	575	18.8%	133
Was the study participant able to twist the cap onto Bottle 1?	98.7%	697	1.3%	9
Did the study participant apply bandage?	91.4%	645	8.6%	61
**Self-Test Procedure**				
Did the study participant shake Bottle 1, four (4) times?^a^	88.9%	627	11.1%	78
Did the study participant pour all the liquid from Bottle 1 into test device?^a^	97.3%	686	2.7%	19
Did participant wait until liquid from Bottle 1 disappeared before adding liquid from Bottle 2 into device?^a^	98.0%	689	2.0%	14
Did the study participant shake Bottle 2, four (4) times?^a^	88.8%	627	11.2%	79
Did the study participant pour the liquid from Bottle 2 into test device?^a^	99.0%	699	1.0%	7
Did participant wait until liquid from Bottle 2 disappeared before adding liquid from Bottle 3 into device?^a^	98.2%	692	1.8%	13
Did the study participant shake Bottle 3, four (4) times?^a^	90.2%	638	9.8%	69
Did the study participant pour the liquid from Bottle 3 into device and wait until liquid disappeared?^a^	97.3%	687	2.7%	19
**Procedure Check**				
Did the participant quit the process at any point?	1.0%	7	99.0%	701
Did the participant perform the steps out of the order?^a^	10.7%	76	89.3%	632
Did the participant miss any step and continued the process despite a missed or incorrect step?	9.5%	67	90.5%	640

^a^Critical step

**Average on all desired outcomes = 91.8%**

**Average on critical steps = 92.4%**

Following the fingerstick blood collection, there were three types of procedures carried out as part of the test process: (1) shaking bottles (accomplished with range of 88.8 to 90.2% of time), (2) pouring contents into the test device (97.3 to 99.0% of participants did so correctly), and (3) waiting for liquids to disappear before adding next liquid into device (participants did this procedure 98.0 to 98.2% of the time). Over 89% (*n* = 632) of participants performed the procedural steps of the self-test in the correct order, while less than 10% (*n* = 67) missed any steps and yet continued the process despite this, and only 1.0% (*n* = 7) quit the process before the test was completed.

Table 6 shows the results from the self-test questionnaire given to each participant. Regarding the instructions for use, 96.7% (*n* = 670) of participants found them easy to follow. In terms of device use, over 90% (*n* = 626) of participants indicated that they were confident with performing the test on their own, and 96.2% (*n* = 666) found the device easy to use. 94.7% (*n* = 655) of participants indicated that they would use the test again and 95.5% (*n* = 658) would recommend this test to a sexual partner or friend. In terms of where participants would prefer using the self-test, 58.6% (*n* = 399) indicated that they would do the test at home, while 41.4% (*n* = 282) indicated that they would prefer to do the test at a clinic.

**Table 6:** Self-Test Questionnaire (n = 708)

Question	Yes		No	
	%	n	%	No
**Instruction Use**				
Did you use the test instructions?	99.4%	689	0.6%	4
Were the instructions for use easy to follow?	96.7%	670	3.3%	23
Were the pictures and illustrations helpful?	99.1%	685	0.9%	6
Was the “NOT FOR USERS” section in the IFU helpful?	44.4%	307	55.6%	385
**Device Use**				
Was the device easy to use?	96.2%	666	3.8%	26
Were you confident with performing this test on your own?	90.6%	626	9.4%	65
**Self-Test Experience**				
Would you use this test again?	94.7%	655	5.3%	37
Would you prefer to use this test at home (yes) or get tested at a clinic (no)?	58.6%	399	41.4%	282
Would you recommend this test to a sexual partner/friend?	95.5%	658	4.5%	31

### Mock test interpretation results

A total of 404 participants volunteered to complete the mock test interpretation (see Table 7 for a full breakdown of mock test results). Overall, 97.8% (*n* = 395) of participants correctly identified a “strong positive”, 90.6% (*n* = 366) a “weak positive”, 99.3% (*n* = 401) a “negative” result, 97.8% (n = 395) an “invalid” (with no control, no test) result, and 93.6% (*n* = 378) an “invalid” (with no control, with test) result.

**Table 7:** Mock Results Interpretation (*n* = 404)

Participant Interpretation	%
**Strong Positive**	
Positive (*n* = 395)	97.8%
Negative (*n* = 3)	0.7%
Invalid (*n* = 5)	1.2%
Do not know (*n* = 1)	0.2%
**Weak Positive**	
Positive (*n* = 366)	90.6%
Negative (*n* = 7)	1.7%
Invalid (*n* = 28)	6.9%
Do not know (*n* = 3)	0.7%
**Negative**	
Positive (*n* = 3)	0.7%
Negative (*n* = 401)	99.3%
Invalid (*n* = 0)	0.0%
Do not know (n = 0)	0.0%
**Invalid (no control, no test)**	
Positive (*n* = 1)	0.2%
Negative (*n* = 2)	0.5%
Invalid (*n* = 395)	97.8%
Do not know (*n* = 6)	1.5%
**Invalid (no control, with test)**	
Positive (*n* = 9)	2.2%
Negative (*n* = 11)	2.7%
Invalid (*n* = 378)	93.6%
Do not know (*n* = 6)	1.5%

## Discussion

Since data from this study was intended to be submitted by the manufacturer to Health Canada for evidence of the INSTI HIV Self-Test safety and effectiveness as part of the device license application, it was important to determine if the self-test performance met published performance targets. The Health Canada Guidance for Manufacturers of Human Immunodeficiency Virus (HIV) Rapid Diagnostic Tests (RDTs) for use at the Point of Care (POC) or for Self-Testing provides performance targets that must be met for clinical sensitivity and specificity: “Evidence that the RDT intended for use at the POC or for Self-Testing has a minimum sensitivity and specificity of ≥99% for HIV antibody detection should be provided” [15]. Primary efficacy analysis with 678 participants who completed the HIV self-testing study revealed a positive percent agreement of 100% (5/5, 95% CI: 43.6–97.0%) and a negative percent agreement of 99.5% (614/617, 95% CI: 98.6–99.8%) when comparing the valid self-tester results to the Abbott Architect. The overall specificity, indicated as negative percent agreement of 99.5%, with the lower bound of the 95% CI at 98.6% meets the required performance criteria.

There was a 93.5% (*n* = 633) concordance in the self-test results interpretation between the untrained self-test study participants and the trained observers for the subjects in the primary efficacy analysis. Most discordant results were in the invalid or “do not know/not sure” (uncertainty) response categories.

A total of 38 participants (5.6% of sample) interpreted their self-test results as invalid, including one participant whose venous blood tested positive with the Abbott Architect. Of these, 22 were also interpreted as invalid by the observers, including the one participant living with HIV; however, 15 were interpreted as negative by the observers indicating there was a visible control dot present in the self-test which was either not observed by the self-tester, or not interpreted correctly. In some of these cases, the control dot intensity was indicated to be faint. A faint, but visible control dot may also have led to “do not know/not sure” interpretations recorded by both the self-testers and observers. Mitigations may be needed to improve the fingerstick blood collection process since usability results indicated that nearly 1 in 5 participants had difficulties in getting a free-flowing blood drop to fall into the INSTI sample diluent (Bottle 1). These mitigations could include revisions to the package insert to provide more clear instructions on the proper use of the lancet and subsequent blood drop collection, as well alerting the self-tester that even a faint dot intensity is considered valid. Consequently, self-test users should be instructed to conduct self-testing in a well-lit area.

There was an observed tendency for some self-testers to wipe their fingertip on the rim of the INSTI Bottle 1 (sample diluent) instead of allowing the blood drop to fall freely into the sample diluent. This was indicated for 60% (*n* = 22/37) of invalid self-test results and for several self-test results that were still valid. A further mitigation to address the critical factor for the success in getting the blood drop into Bottle 1 could be to revise the self-test package insert to indicate, with illustration, that one should not scrape or wipe the blood into Bottle 1.

The mock results interpretations for the 404 participants who volunteered to participate in this portion of the study show a high concordance with the expected results. The percent agreement with expected results ranged from 90.6% (*n* = 366) for the weak positive result to 99.3% (*n* = 401) for the negative result, indicating that untrained self-testers can successfully interpret a range of self-test results including strong positive, weak positive, negative, and invalid test results. However, mitigations to the package insert to call attention to the possibility of faint dot intensity could improve the lower success rate in interpreting weak positive results.

In general, participants expressed a high level of satisfaction with the HIV-ST experience as shown in Table 6: 96.7% (*n* = 670) of participants found the instructions for use easy to follow and 96.2% (*n* = 666) found the self-test device easy to use. 94.7% (*n* = 655) of participants would use the INSTI HIV Self-Test again, and 95.5% (*n* = 658) would recommend its use to a sexual partner or friend. Of interest and for consideration – approximately 60% (*n* = 399) of participants indicated a preference for using the self-test in a home environment while 40% (*n* = 282) would prefer to use the test at a clinic or health care facility, suggesting that both assisted (supervised) and unassisted self-testing strategies will likely be needed for “real world” access and uptake.

Overall, acceptance of the INSTI HIV Self-Test by intended users in this study was high, and similar to studies that reported that oral fluid-based HIV self-testing is highly acceptable among a variety of populations [7, 16, 17]. Although data on use of blood-based HIV self-tests remains scarce [18–20], performance, usability and acceptance in this study were similar to an INSTI HIV Self-Test study conducted in Kenya [21]. This suggests that populations in both settings were able to conduct and perform the blood-based test as per instructions and agreement and performance metrics were aligned. This attests to the broad use of blood based self-tests as an alternative to oral self-tests.

## Conclusions

The impact of the COVID-19 pandemic and its related public health control measures on HIV treatment and prevention, including access to facility-based testing, has had profound effects on individuals, communities, and societies across the world [22]. Implementation of HIV self-testing has been shown to increase HIV testing rates in men who have sex with men [23], and we found high acceptability amongst the diverse sample of participants in the current study. So, the addition of this newly approved HIV self-test into the Canadian HIV testing landscape could have similar outcomes. An approved blood-based HIV self-test in Canada can offer an accurate, acceptable and simple alternative to facility-based HIV testing for key populations, particularly when impacted by COVID-19 pandemic restrictions. Efforts to mitigate the frequency of invalid results, such as improved instructions for collection of the fingerstick blood sample, should be considered.

## Supplementary Information

**Additional file 1.** Appendix A – Inclusion and Exclusion Criteria; Appendix B – Exclusion List; Appendix C – Survey Instrument.

## Data Availability

The datasets used and/or analyzed during the current study are available from the corresponding author on reasonable request.

## Abbreviations

HIV: Human Immunodeficiency Virus
CM: Comparator Methods
PPA: Positive Percent Agreement
NPA: Negative Percent Agreement

## References

[1] Public Health Agency of Canada. Estimates of HIV incidence, prevalence and Canada’s progress on meeting the 90-90-90 HIV targets, 2018: Government of Canada; 2020 [Available from: https://www.canada.ca/en/public-health/services/publications/diseases-conditions/summary-estimates-hiv-incidence-prevalence-canadas-progress-90-90-90.html.

[2] Public Health Agency of Canada. Government of Canada announces progress and new investments to eliminate HIV/AIDS as a public health threat: Government of Canada; 2016 [Available from: https://www.canada.ca/en/public-health/news/2016/12/government-canada-announces-progress-new-investments-eliminate-aids-public-health-threat.html.

[3] Public Health Agency of Canada. Summary: Estimates of HIV incidence, prevalence and Canada’s progress on meeting the 90-90-90 HIV targets, 2016: Government of Canada; 2018 [Available from: https://www.canada.ca/en/public-health/services/publications/diseases-conditions/summaryestimates-hiv-incidence-prevalence-canadas-progress-90-90-90.html.

[4] Elliott T, Sanders EJ, Doherty M, Ndung'u T, Cohen M, Patel P, Cairns G, Rutstein SE, Ananworanich J, Brown C, Fidler S (2019). Challenges of HIV diagnosis and management in the context of pre-exposure prophylaxis (PrEP), post-exposure prophylaxis (PEP), test and start and acute HIV infection: a scoping review. J Int AIDS Soc.

[5] World Health Organization. Policy brief: WHO recommends HIV self-testing 2016 [Available from: https://apps.who.int/iris/bitstream/handle/10665/251549/WHO-HIV-2016.21-eng.pdf;jsessionid=D6B8C7E8DA3CB8F2D43C3C6882088629?sequence=1.

[6] Unitaid WHO. Market and technology landscape: HIV rapid diagnostic tests for self-testing 2018 [Available from: https://unitaid.org/assets/HIVST-landscape-report.pdf.

[7] Johnson C, Baggaley R, Forsythe S, Van Rooyen H, Ford N, Mavedzenge SN (2014). Realizing the potential for HIV self-testing. AIDS Behav.

[8] Pai N, Karellis A, Kim J, Peter T (2020). Modern diagnostic technologies for HIV. Lancet HIV.

[9] Witzel T, Bourne A, Burns F, Rodger A, McCabe L, Gabriel M, Gafos M, Ward D, Collaco-Moraes Y, Dunn DT, Speakman A, Bonell C, Pebody R, Lampe FC, Harbottle J, Phillips AN, McCormack S, Weatherburn P (2020). HIV self-testing intervention experiences and kit usability: results from a qualitative study among men who have sex with men in the SELPHI (self-testing public health intervention) randomized controlled trial in England and Wales. HIV Med.

[10] Kalibala S, Tun W, Cherutich P, Nganga A, Oweya E, Oluoch P (2014). Factors associated with acceptability of HIV self-testing among health care workers in Kenya. AIDS Behav.

[11] Pai N, Smallwood M, Desjardins L, Goyette A, Birkas KG, Vassal A-F (2018). An unsupervised smart app–optimized HIV self-testing program in Montreal, Canada: cross-sectional study. J Med Internet Res.

[12] Pai N, Bhargava M, Joseph L, Sharma J, Pillay S, Balram B, et al. Will an unsupervised self-testing strategy be feasible to operationalize in Canada? Results from a pilot study in students of a large Canadian university. AIDS Res Treatment. 2014;2014. 10.1155/2014/747619 PMC3912878 24511392

[13] Pai N, Smallwood M, Gulati D, Lapczak N, Musten A, Gaydos C (2018). What do key stakeholders think about HIV self-testing in Canada? Results from a cross-sectional survey. AIDS Behav.

[14] Majam M, Mazzola L, Rhagnath N, Lalla-Edward ST, Mahomed R, Venter WDF, Fischer AE (2020). Usability assessment of seven HIV self-test devices conducted with lay-users in Johannesburg. South Africa Plos one.

[15] Health Canada. Guidance Document - Guidance for Manufacturers of Human Immunodeficiency Virus (HIV) Rapid Diagnostic Tests (RDTs) for use at the Point of Care or for Self-Testing: Government of Canada; 2017 [Available from: https://www.canada.ca/content/dam/hc-sc/migration/hc-sc/dhp-mps/alt_formats/pdf/md-im/applic-demande/guide-ld/human-immunodeficiency-virus-rapid-diagnostic-tests-guidance.pdf.

[16] Pai N, Sharma J, Shivkumar S, Pillay S, Vadnais C, Joseph L (2013). Supervised and unsupervised self-testing for HIV in high-and low-risk populations: a systematic review. PLoS Med.

[17] Figueroa C, Johnson C, Verster A, Baggaley R (2015). Attitudes and acceptability on HIV self-testing among key populations: a literature review. AIDS Behav.

[18] World Health Organization. WHO Prequalification of In Vitro Diagnostics Programme. Product: SURE CHECK HIV self-test 2019 [Available from: https://www.who.int/diagnostics_laboratory/evaluations/pq-list/191129_pqdx_0054_006_01_sure_check_hiv_self_test.pdf?ua=1.

[19] World Health Organization. WHO Prequalification of In Vitro Diagnostics Programme. Product: Mylan HIV self-test 2019 [Available from: https://www.who.int/diagnostics_laboratory/evaluations/pq-list/191003_amended_pqpr_0320_090_00_mylan_hiv_self_test_v2.pdf?ua=1.

[20] World Health Organization. WHO Prequalification of In Vitro Diagnostics Programme. Product: INSTI HIV self-test 2019 [Available from: https://www.who.int/diagnostics_laboratory/evaluations/pq-list/191111_amended_final_pqpr_0002_002_01_selftest_v3.pdf?ua=1.

[21] Bwana P, Ochieng’ L, Mwau M. Performance and usability evaluation of the INSTI HIV self-test in Kenya for qualitative detection of antibodies to HIV. PloS one. 2018, 13;(9):e0202491. 10.1371/journal.pone.0202491 PMC6136890 30212525

[22] Chenneville T, Gabbidon K, Hanson P, Holyfield C (2020). The impact of COVID-19 on HIV treatment and research: a call to action. Int J Environ Res Public Health.

[23] Zhang C, Li X, Brecht M-L, Koniak-Griffin D (2017). Can self-testing increase HIV testing among men who have sex with men: a systematic review and meta-analysis. PLoS One.

