# Supplementary material for: An observed, prospective field study to evaluate the performance and acceptance of a blood-based HIV self-test in Canada
Source: BMC Public Health. 2021 Jul 18;21:1421. doi: 10.1186/s12889-021-11418-z (PMC8286440; doi:10.1186/s12889-021-11418-z)
Supplement: Supplementary file 1 — Additional file 1. Appendix A – Inclusion and Exclusion Criteria; Appendix B – Exclusion List; Appendix C – Survey Instrument. [file 12889_2021_11418_MOESM1_ESM.docx]

# **Appendix A – Inclusion and Exclusion Criteria**

Inclusion criteria:

- Participants presenting for voluntary testing for HIV infection in the clinic or community-based setting
- Able to understand and complete study informed consent; able to sign or instruct the study research coordinator to sign the informed Consent form
- Able to complete required testing procedures in one session on the allocated testing day;
- Able to speak, read, and write in English or French
- Person is ≥18 years of age
- Willingness to participate in the study site’s standard of care HIV counselling and testing program and receive the study site’s standard of care test results
- Willingness to participate in providing the necessary volume of whole blood collected through venous blood draw (approximately 7 ml) and finger stick for use in the study protocol testing methods

Exclusion criteria:

- Are known HIV positive
- Do not meet the inclusion criteria
- Have received any experimental HIV vaccine
- Are currently on a PrEP regimen
- Have participated in any prior, or concurrent trial of HIV self-tests
- A practicing medical healthcare professional (doctor, nurse or HIV Counsellor that performs HIV testing with Rapid Tests)
- Has used an RDT for self-testing previously

# **Appendix B – Exclusion List**

| **Study Exclusion List (n=89)** |
| --- |
| **EXCLUSIONS FROM ALL ANALYSES (n=47)** |
| **1. Missing PID in Qualtrics (n=22)** |
| ON01-007, 052, 190, 259, 272, 289, 303, 304, 316, 317, 318, 331, 343, 350, 358, 380 |
| ON02-061, 064, 070, 071, 084 |
| QC01-046 |
|  |
| **2. Result of enrollment: withdrawn (n=2)** |
| ON01-026, 027 |
|  |
| **3. No self-test/mock test (n=23)** |
| ON01-019, 150, 274, 287, 308, 334 |
| ON02-003, 004, 013, 031, 032, 038, 044, 046, 050, 054, 068, 075, 088, 090, 094 |
| MB01-113 |
| QC01-007 |
|  |
| **EXCLUSIONS FROM PRIMARY EFFICACY AND USABILITY ANALYSES (n=12)** |
| **4. No self-test result (n=12)** |
| ON01-051, 054, 055, 056, 116, 157, 197 |
| ON02-052 |
| MB01-137 |
| QC01-011, 064, 109 |
|  |
| **FURTHER EXCLUSIONS FROM PRIMARY EFFICACY ANALYSES (n=30)** |
| **5. No self-test result (complete self-test data but no participant interpretation) (n=9)** |
| ON01-001, 002, 004, 013, 016, 025, 028, 037, 046 |
|  |
| **6. No lab result (n=9)** |
| ON01-011, 320, 336, 341, 362, 395, 397 |
| ON02-009, 012 |
|  |
| **7. Poured bottle 1 into bottle 2 before adding to test device (n=12)** |
| ON01-336, 355, 368, 369, 377, 370, 371, 372, 390, 398 |
| ON02-081, 086 |
| Note: Coding for Sites |
| ON01: Hassle Free Clinic |
| ON02: Women's Health in Women's Hands |
| MB01: Nine Circles Community Health Centre |
| QC01: Clinique Médicale l'Actuel |

# **Appendix C – Survey Instrument**

1. **Enrolment Questionnaire**
2. **Participant Demographics**

| Initials: | | | | | |
| --- | --- | --- | --- | --- | --- |
| Gender: | | | | | |
| Year of Birth: **YYYY** | | | | | |
| Age: | 18-25 | 26-35 | 36-45 | 46-55 | >55 |
| Apply the Participant ID Number Label here: …………………………………………….. | | | | | |

1. **Inclusion Criterion**

Participants can be included in the study if they meet **all** of the following criteria:

| Understands and signs or instructs the Research Assistant (observer) to sign the informed Consent form |  |
| --- | --- |
| Able to complete the required testing on the allocated testing day |  |
| Able to speak / read / write English or French |  |
| Is ≥18 years of age |  |
| Willingness to provide the necessary volume of whole blood collected through venous blood draw (approximately 7 ml) and finger stick for use in the study protocol testing methods. |  |
| Willingness to participate in the study site’s standard of Care HIV counseling and testing program and receive the study site’s standard of care test results |  |

1. **Exclusion Criterion**

Participant will be excluded from the study if they meet **any** of the following criterion:

| Are known HIV positive |  |
| --- | --- |
| Do not meet any of the inclusion criteria |  |
| Has received any experimental HIV vaccine |  |
| Is currently on a PrEP regimen |  |
| Have participated in any prior, or concurrent trial of HIV self-tests |  |
| Are a practicing medical healthcare professional (doctor, nurse or HIV Counsellor that performs HIV testing with Rapid Tests) |  |
| Has used an RDT for self-testing previously |  |
| Any condition which, in the opinion of the RA, would make the participant unsuitable or unsafe for enrolment or could interfere with the completion of the assessment, consent form and questionnaire etc. or bias the outcome, i.e. being unable to see / read by forgetting to bring reading glasses, being intoxicated or acute sickness, or visibly distressed.  Record the reason for exclusion: | |

Note to the Observer: There is no need to complete the remaining questions if any of the exclusion criterion applies.

1. **Participant Race/Ethnicity**

| White |  |
| --- | --- |
| Black |  |
| First Nation, Métis, Inuit |  |
| South Asian  (e.g., East Indian, Pakistani, Sri Lankan, Punjabi, Bangladeshi, Nepali) |  |
| Southeast Asian  (e.g., Chinese, Japanese, Vietnamese, Cambodian, Indonesian, Korean, Filipino) |  |
| Arab/West Asian  (e.g., Armenian, Egyptian, Iranian, Lebanese, Moroccan) |  |
| Latin American  (e.g., Mexican, Central/South American) |  |
| Other - includes mixed ethnicity; specify: |  |
| Country of birth |  |

1. **Participant Background information**
2. Employment status

| Employed |  |
| --- | --- |
| Unemployed |  |

1. Dominant hand

| Right |  |
| --- | --- |
| Left |  |

1. Visual status (use of reading glasses)

| Yes |  |
| --- | --- |
| No |  |

1. Highest education level

| Primary |  |
| --- | --- |
| Secondary (High School) |  |
| College |  |
| University or higher |  |

1. Reading/writing impairment

| Yes |  |
| --- | --- |
| No |  |

1. **Medical Conditions and Risk Category Checklist**
2. Experience with HIV testing

Yes  No

If Yes, approximate date of last HIV Test: **YYYY-MMM-DD**

1. Self-reported HIV status:

| **PLEASE TICK THE APPROPRIATE OPTION BELOW** | |
| --- | --- |
| Unknown status/never been tested |  |
| Negative status |  |
| Positive status (if positive, then apply the exclusion criterion) |  |

1. Self-reported medical conditions:

| **PLEASE TICK THE APPROPRIATE OPTION BELOW** | |
| --- | --- |
| Diabetes |  |
| Hypertension |  |
| Visual impairment |  |
| Existing/Recent Sexually transmitted diseases (example: Chlamydia, Gonorrhea, etc.) |  |
| Other |  |
| List which other conditions: |  |

1. Participant is pregnant: Yes  No  N/A
2. Self reported risk category

| **PLEASE TICK ALL THAT APPLY BELOW** | |
| --- | --- |
| Unprotected sex with men |  |
| Unprotected sex with women |  |
| Multiple sexual partners |  |
| Injection drug user |  |
| Born to an HIV positive mother |  |
| Sexual partner is HIV positive |  |
| Sexual partner is a bisexual male |  |
| Other |  |

**Result of Enrolment Questionnaire:**  Enrolled  Excluded  Withdrawn

Please record the reason for Withdrawal if applicable: __________________________________________________________________

1. **Observer’s Data Collection Form**

(The Research Assistant(observer) will complete this form based on their observation of the self tester)

| **Device type: INSTI HIV Self Test** | **Researcher:** | **Date** *(dd/MMM/YYYY*)**:** |
| --- | --- | --- |
| Apply the Participant ID Number Label here: ………………………………………….  **___ ___ ___** | | |
| **Start time** (Time when the participant opened the test pouch)**:** | | |
| **End time** (Time when the participant read the result)**:** | | |

PART A. Observations about the Participant

1. Did the study participant read/use the Instructions for Use (IFU)?

Yes

No

If yes, was the IFU read before the test?

Yes

No

Was it referred to during the test process?

Yes

No

1. Did the study participant wash and dry their hands as instructed in the IFU?

Yes

No

1. Was it difficult for the study participant to remove the test device from the pouch?

Yes

No

If Yes, describe what seemed to be the problem? (E.g. could not find tear point, weakness,confusion) _______________________________________________________________________________________________________________________________________________________________________________________________________________

1. Was the study participant able to remove the cap of Bottle 1?

Yes

No

If No, describe what seemed to be the problem? _______________________________________________________________________________________________________________________________________________________________________________________________________________

1. Did the study participant twist the tip of the lancet off?

Yes

No

If No, describe what seemed to be the problem? _______________________________________________________________________________________________________________________________________________________________________________________________________________

1. Did the study participant rub his/her finger correctly (up and down/vertical motion)?

Yes

No

If No, describe what seemed to be the problem?

_______________________________________________________________________________________________________________________________________________________________________________________________________________

1. Was the study participant able to lance his/her finger correctly?

Yes

No

If No, describe what seemed to be the problem? _______________________________________________________________________________________________________________________________________________________________________________________________________________

1. Was the study participant able to form a blood droplet?

Yes

No

If No, describe what seemed to be the problem? _______________________________________________________________________________________________________________________________________________________________________________________________________________

1. Was the study participant able to get the blood droplet to fall into Bottle 1?

Yes

No

If No, then how was it done? _______________________________________________________________________________________________________________________________________________________________________________________________________________

1. Was the study participant able to twist the cap onto Bottle 1?

Yes

No

If No, then how was it done?

__________________________________________________________________________________________________________________________________________

1. Did the study participant apply bandage?

Yes

No

1. Did the study participant shake Bottle 1, 4 times?

Yes

No

If No, then how many times
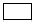


1. Did the study participant pour all the liquid from Bottle 1 into test device?

Yes

No

If No, then how was it done?

_______________________________________________________________________________________________________________________________________________________________________________________________________________

1. Did the study participant pour the liquid from Bottle 1 into Bottle 2 before adding it to the test device?

Yes

No

If YES, please describe

_______________________________________________________________________________________________________________________________________________________________________________________________________________

1. Did the study participant wait until liquid from Bottle 1 was disappeared before adding liquid from Bottle 2 into the test device?

Yes

No

If No, then how was it done?

_______________________________________________________________________________________________________________________________________________________________________________________________________________

1. Did the study participant shake Bottle 2, 4 times?

Yes

No

If No, then how many times?
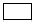


1. Did the study participant pour the liquid from Bottle 2 into test device?

Yes

No

If No, then how was it done?

_______________________________________________________________________________________________________________________________________________________________________________________________________________

1. Did the study participant wait until liquid from Bottle 2 disappeared before adding liquid from Bottle 3 into the test device?

Yes

No

If No, then how was it done?

_______________________________________________________________________________________________________________________________________________________________________________________________________________

1. Did the study participant shake Bottle 3, 4 times?

Yes

No

If No, then how many times?
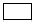


1. Did the study participant pour the liquid from Bottle 3 into device and wait until liquid disappeared?

Yes

No

If No, then how was it done?

_______________________________________________________________________________________________________________________________________________________________________________________________________________

1. Did the participant quit the process at any point? If Yes, explain

__________________________________________________________________________________________________________________________________________

1. Did the participant perform the steps out of the order? i.e. did they add the solutions in the incorrect order?

__________________________________________________________________________________________________________________________________________

1. Did the participant miss any step and continued the process despite a missed or incorrect step?

__________________________________________________________________________________________________________________________________________

1. What was the participant’s apparent level of stress?

Calm

Appears anxious

Verbally communicates distress

Staff intervention required

Any other observer comments:

NOTES: (Was there significant hesitation or indecision at specific steps or overall? Did they say anything; did they ask any questions of the interviewer during the process? Which steps were skipped or modified?) _______________________________________________________________________________________________________________________________________________________________________________________________________________

_____________________________________________________________________

PART B Observer’s Interpretation of the Participant’s INSTI HIV Self Test Result

1. Is the **control** dot present?

Yes  No

_________________________________________________________________

1. Is the **test** dot present?

Yes  No

__________________________________________________________________

1. Is the test impossible to read? (if Yes, explain why)

Yes  No

_____________________________________________________________________

1. Observer’s interpretation of INSTI result

Negative

Positive

Invalid/test did not work

Do not know/Not sure

Could not complete INSTI HIV self test

1. Participant’s interpretation of INSTI result

Negative

Positive

Invalid/test did not work

Do not know/Not sure

Could not complete INSTI HIV self test

**Additional Interpretation Comments:**

_________________________________________________________________________________________________________________________________________________________________________________________________________________________________

1. **Participant’s INSTI HIV Self Test Result Record Form**

(The study participant will complete this form for recording their own INSTI HIV Self Test result)

| **Device: INSTI HIV Self Test** | **Date** *(dd/MMM/YYYY*)**:** |
| --- | --- |
| Apply the Participant ID Number Label here: …………………………………………. | |

1. What is the INSTI HIV Self Test result?

Negative

Positive

Invalid/Test did not work

Do not know/Not sure

Comments (if any): ______________________________________________________________

1. Is the test impossible to read?

Yes  No

If Yes, explain why: ___________________________________________________

1. **Self-Test Questionnaire**

(The Research Assistant (observer) to ask these questions to the self tester and complete the questionnaire with the self-tester’ answers.)

| **Device type:**  **INSTI HIV Self Test** | **Researcher:** | **Date** (*dd/MMM/YYYY*): |
| --- | --- | --- |
| Apply the Participant ID Number Label here: …………………………………………. | | |

1. Did you use the test instructions?

Yes  No

If No, please explain _________________________________________________________________________________________________________________________________________________________________________________________________________________________________

1. Were the Instructions for Use (referred to as IFU) easy to follow?

Yes  No

If No, please explain

______________________________________________________________________________________________________________________________________________________

1. Were the pictures and illustrations helpful?

Yes  No

If No, please explain

______________________________________________________________________________________________________________________________________

1. Were the “NOT FOR USERS” section in the IFU helpful?

Yes  No

If No, please explain

____________________________________________________________________________________________________________________________________________

1. Please look at the IFU in front of you, and show me any part which you did not read?______________________________________________________
2. Please look at the IFU and show me which part was hard to understand?

_____________________________________________________

1. Was the device easy to use?

Yes  No

If No, please explain the steps that were difficult or confusing

______________________________________________________________________________________________________________________________________________________

1. Were you confident with performing this test on your own?

Yes  No

If No, please explain why you were not? _________________________________________________________________________________________________________________________________________________________________________________________________________________________________

1. What should you do if you have a negative result?

_________________________________________________________________________________________________________________________________________________________________________________________________________________________________

1. What should you do if you have a positive result?

_________________________________________________________________________________________________________________________________________________________________________________________________________________________________

1. What should you do if you have an invalid result?

_________________________________________________________________________________________________________________________________________________________________________________________________________________________________

1. What should you do if you are not sure of your result?

_________________________________________________________________________________________________________________________________________________________________________________________________________________________________

1. Would you use this test again?

Yes  No

If No, please explain why ______________________________________________________________________________________________________________________________________________________

1. Would you prefer to use this test at home or get tested at a clinic?

Home  Clinic

Please explain your choice

______________________________________________________________________________________________________________________________________________________

1. Would you recommend this test to a sexual partner/friend?

Yes  No

If No, please explain why

______________________________________________________________________________________________________________________________________________________

1. Do you have suggestions on how to make this product easier and the IFU better to use? Please point to anything specific on the IFU to assist.

____________________________________________________________________________________________________________________________________________________________________________________________________________________________________________________________________________________________________________

1. Do you have any other comments about the test or your experience with self-testing?

____________________________________________________________________________________________________________________________________________________________________________________________________________________________________________________________________________________________________________

1. **Participant’s Mock INSTI Result Interpretation Record Form**

(The Observer will complete this form for recording the mock INSTI HIV Self Test result interpretation)

| **Device: INSTI HIV Self Test (Mock Test Results)** | **Date** *(dd/MMM/YYYY*)**:** |
| --- | --- |
| Apply the Participant ID Number Label here: …………………………………………. | |

Note to the Observer: For each of the following contrived membrane units presented to the participant, record the participant’s result interpretation as expressed by the participant:

| **Test Device** | **True Mock Test Result**  (do not share this with the participant) | **Participant’s Interpretation of the Mock Test Result**  (ask the participant their interpretation of the mock test result) | **Does the participant’s interpretation match the true mock result?** |
| --- | --- | --- | --- |
| 1 | Strong HIV Positive | strong positive  weak positive  negative  invalid (no control or test spot is visible)    invalid (the control dot is not visible but the test dot is visible)  don’t know/not sure or quit the process | Yes  No |
| 2 | Weak HIV Positive | strong positive  weak positive  negative  invalid (no control or test spot is visible)    invalid (the control dot is not visible but the test dot is visible)  don’t know/not sure or quit the process | Yes  No |
| 3 | HIV Negative | strong positive  weak positive  negative  invalid (no control or test spot is visible)    invalid (the control dot is not visible but the test dot is visible)  don’t know/not sure or quit the process | Yes  No |
| 4 | Invalid (no control or test spot is visible) | strong positive  weak positive  negative  invalid (no control or test spot is visible)    invalid (the control dot is not visible but the test dot is visible)  don’t know/not sure or quit the process | Yes  No |
| 5 | Invalid (the control dot is not visible but the test dot is visible) | strong positive  weak positive  negative  invalid (no control or test spot is visible)    invalid (the control dot is not visible but the test dot is visible)  don’t know/not sure or quit the process | Yes  No |
